# Surgical Correction of Ankyloglossia Using Diode Laser-Assisted Frenectomy in a Pediatric Patient: A Case Report

**DOI:** 10.7759/cureus.62024

**Published:** 2024-06-09

**Authors:** Prachi M Rakhunde, Dhruvi Solanki, Punit Fulzele, Rashi Dubey, Ramakrishna Yeluri, Sakshi P Kabra

**Affiliations:** 1 Department of Dentistry, Sharad Pawar Dental College, Datta Meghe Institute of Higher Education and Research, Wardha, IND; 2 Department of Pedodontics and Preventive Dentistry, Sharad Pawar Dental College, Datta Meghe Institute of Higher Education and Research, Wardha, IND

**Keywords:** tongue, lingual frenum, case report, laser, feeding difficulty, speech problem, pediatric patient, tongue-tie, diode-laser, ankyloglossia

## Abstract

Tongue-tie is a continuation of the lingual frenum that is attached to the tip of the tongue. It is a congenital oral anomaly that could restrict tongue movements, caused by a lingual frenum a membrane that originates from the floor of the mouth to the bottom of the tongue that is too thick and short, which limits the natural ability of the tongue to move and function. The tongue is an auxiliary organ that facilitates speaking, mastication, and deglutition. This condition may result in several difficulties including chewing, breastfeeding, speech, and pronunciation of particular words, as well as possessing social and mechanical consequences. Ankyloglossia can be seen in young age groups. The use of lasers has increased in dentistry in recent years. However, in oral and maxillofacial surgery, the use of lasers has been largely restricted to soft tissues, and less focus is placed on the use of hard tissues. Carbon dioxide (CO_2_) lasers, erbium-doped yttrium aluminum garnet (Er: YAG) lasers, and Er, the erbium, chromium: yttrium: scandium gallium-garnet (Cr: YSGG) lasers are among the several types of lasers that have been utilized in dentistry for correction of soft tissues as well as for hard tissues.

## Introduction

The word ankyloglossia is derived from the Greek terms "agkilos" meaning curved and "glossa" meaning tongue. During the year 1960, Wallace described ankyloglossia or tongue-tie as a disorder wherein a small soft tissue attachment, called frenulum prevents the tongue tip from protruding past the lower incisor teeth [1-3]. Tongue-tie affects 2-20% of children, with more preference found in infants (7%) than in children [4]. The male-female ratio of ankyloglossia is 2.5:1. It is related to a few uncommon syndromes, including lip pit syndrome, X-linked cleft palate syndrome, and Smith-Lemli-Opitz syndrome (SLOS) [4-6].

The aberrant lingual frenum can be treated by procedures such as frenectomy or frenotomy. A frenotomy includes an incision and relocation of the frenal connection, but a frenectomy is the entire removal of the frenum, with its attachment to an underlying bone [7]. A frenectomy can be performed by light amplification by stimulated emission of radiation (LASER), electrosurgery, or the standard scalpel approach. According to the reports, the use of LASERs during lingual frenectomies is a safe and successful technique that has the benefits of less discomfort, little or no swelling and scarring, quicker recovery times, ease of use, and decreased post-operative infections [8].

A laser has been proven to be an efficient and painless method of treatment. In recent years neodymium-doped yttrium aluminum garnet (Nd: YAG), diode, or erbium lasers have taken the lead over conventional scalpel surgery. Diode laser works at the wavelength of 810-1064 nm. Basically, they are preferred for soft tissue surgeries. Laser works on the principle of photothermal interaction with the tissues. The radiant light energy emitted by the laser is absorbed by the targeted tissues and it is then transformed into thermal energy which changes the tissue structure and also leads to coagulation [9,10]. Carbon dioxide lasers, erbium-doped yttrium aluminum garnet (Er: YAG) lasers, and the erbium, chromium: yttrium: scandium gallium-garnet (Er, Cr: YSGG) lasers are among the several types of lasers that have been utilized in dentistry for correction of soft tissues as well as for hard tissues [11].

## Case presentation

**Figure 1:**
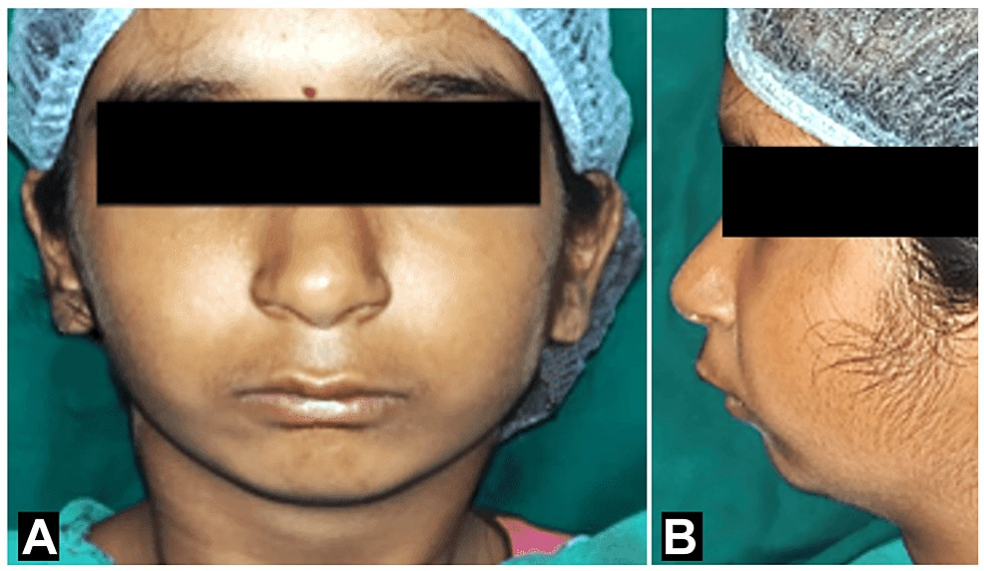
Extra-oral images - (A) frontal profile showing deficient mandible and (B) lateral profile showing convex profile.

A 13-year-old female patient reported in the outpatient department of pediatric and preventive dentistry with the chief complaint of slurred speech along with a complaint of malaligned teeth in the upper and lower anterior teeth region of the jaw. Extra oral examination showed a convex facial profile with potentially incompetent lips due to a retruded mandible (Figures 1A, 1B). The patient had undergone the surgery of patent ductus arteriosus at the age of two and a half years.

**Figure 2:**
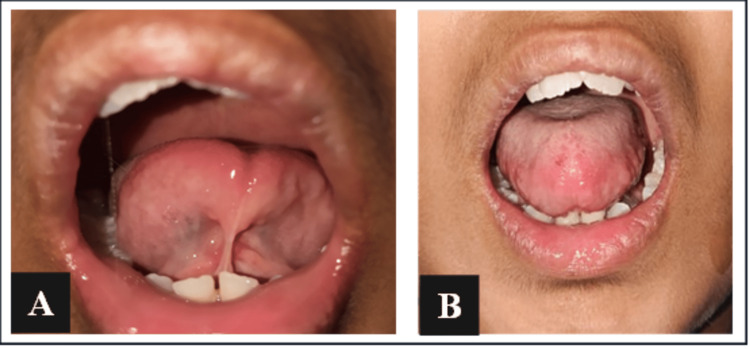
Pre-operative images - (A) elevated tongue showing heart-shaped tip and (B) inability to protrude the tongue.

On intraoral examination, class II molar relation on both sides with V-arched palate was observed along with the presence of tongue-tie. Overjet was increased. All the permanent teeth were present except for bilateral maxillary permanent lateral incisors. Deciduous maxillary lateral incisors were over-retained and the lower left permanent mandibular canine was rotated mesiolingually. When the tongue was elevated towards the palate, it formed a heart-shaped pattern and clefting on the protrusion. As per Kotlow's classification, the case was identified as class III ankyloglossia (Figures 2A, 2B).

A laser-assisted frenectomy was planned using a 980 nm diode laser. Local infiltration of lignocaine was done adjacent to the frenum after spraying it with topical spray. The patient was prepared for the procedure and was made to wear goggles to avoid laser radiation. Protective goggles were also worn by the operator and the assistant. BIOLASE laser was initiated before the surgery and the surgical laser tip of 400 µm was used. The incision was started by keeping the laser tip in contact with the frenal attachment near the attached gingiva of the central incisors.

**Figure 3:**
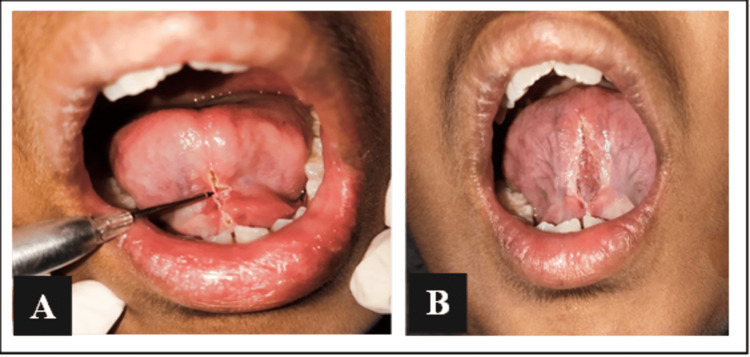
Intra-operative images - (A) laser ablation of lingual frenulum and (B) increased mobility of tongue.

The incision was placed at the midway of the frenum to break the fibrous bands attaching the tongue to the floor of the mouth. The horizontal movement of the laser was done to bisect the longitudinal fibrous bands, giving the operated area a rhomboidal appearance. To seal the margins and remove the overhanging tissues, vertical strokes were placed. No bleeding was observed during the procedure and sutures were not placed (Figures 3A, 3B).

**Figure 4:**
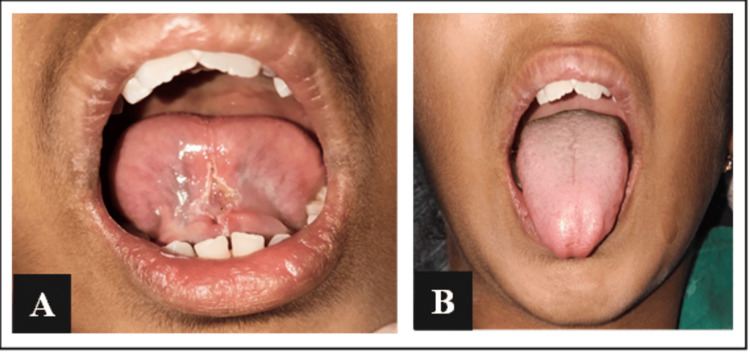
Post-operative images - (A) healing of the surgical site and (B) increased degree of protrusion of the tongue.

Post-operative instructions were given to avoid hard and fried food, hot meals, or any beverages throughout the week, and maintaining optimal oral hygiene practices was advised. Also, post-operative tongue exercises were demonstrated to the patient and was advised to continue till the next recall visit. No medications were advised to the patient and she was recalled after a week for follow-up. After proper healing was noted, the patient was referred to the department of orthodontics and dentofacial orthopedics for correction of the malaligned teeth and retruded mandible (Figures 4A, 4B).

## Discussion

The tongue being the most powerful muscular organ of the oral cavity, influences the growth and development of the dental arches along with the positioning of the teeth. It is also important for performing various functions like speech and deglutition. There exists a framework of tissues known as the lingual frenulum at the ventral surface of the tongue. With the help of it, the tongue is attached through the midline of its inferior surface to the floor of the mouth [12]. For Dentists, diagnosing tongue-tie or ankyloglossia presents a hurdle as most of the focus is imparted to the hard tissue structure in the mouth, i.e., the teeth. It is a congenital disorder that affects roughly 5% of people [13,14]. Getting a correct diagnosis is crucial before undergoing surgery. Speech disorders and tongue-ties are positively correlated [15]. Children below the age of three should have a routine oral examination to properly diagnose tongue-tie. Dentists frequently postpone treating patients with short lingual attachment unless they are having trouble speaking or moving their tongue [12].

Ankyloglossia is defined as a condition that causes the inferior frenum to adhere to the base of the tongue which subsequently limits the ability of the tongue for free movements. There are two different types as follows: partial (called when the lingual frenum is short) and total (fusion of the tongue and the floor of the mouth). In many individuals, ankyloglossia is asymptomatic. However, it may limit the movement of the tongue, and in some cases, recurrent tongue biting is also reported. Patients suffering from tongue-tie have difficulty pronouncing words like ch, t, s, n, d, zh, and th. It results in retrognathism of the mandible due to a lack of the muscular pressure exerted by a normal tongue, thereby giving the patient a convex profile. In severe cases, it might also cause gingival recession of the lower incisors [12,14,16].

Pressure over the frenum allows one to visually detect the aberrant frenum by observing the shifting of the papillary tip as well as the blanching that results from localized ischemia. Placek et al. in 1974 classified frenal attachments as mucosal, gingival, papillary, and papilla penetrating (Table 1) [17].

**Table 1:** Classification of frenal attachment. The table was created by the authors of this study according to the classification of frenal attachment given by Placek et al. in 1974, wherein four types of fiber insertion positions were explained [17].

Type	Position of frenal fibers attachment
Mucosal	Up to mucogingival junction
Gingival	Within the gingiva
Papillary	Penetrating the interdental papilla
Papilla penetrating	Reach the palatine papilla after passing via the alveolar process

Kotlow in 1999 gave a classification for proper assessment of tongue-tie, in which the free margin of the tongue greater than 16 mm was classified as normal. Class I or mild ankyloglossia had a free margin of 12-16 mm. The 8-11 mm free margin is classified under class II or moderate ankyloglossia. Free tongue margin in the range of 3-7 mm is severe (class III) and less than 3 mm is complete ankyloglossia (class IV) [18].

It is well acknowledged that for pediatric frenectomies, surgical lasers are a great and reliable substitute for more conventional scalpel or blade techniques [19]. Although oral soft tissues can be treated with lasers of any wavelength, the choice of a particular therapeutic laser for pediatric oral surgery is determined by the tissue's chromophores, such as collagen, melanin, or hemoglobin, as well as by the photothermal interaction of lasers and optical absorbability for water [20].

Specifically, when applied to vascular lesions rich in hemoglobin, diode, Nd: YAG, and CO_2_ have outstanding properties during cutting of soft tissue or placing incision or contouring, early hemostasis is achieved, and wound disinfection; consequently, sutures are rarely required in these procedures [21].

Post-operative exercises play a vital role in curing patients with speech difficulty due to tongue-tie. Speech and tongue exercises prove to be very effective in such cases as they help in molding the tongue [22]. For children less than six years of age, specialists along with pediatricians must be concerned. After treatment, the patient should be advised for speech therapy to evaluate the proper movement of the tongue as before the treatment the tongue was adaptive to the lower position of the mouth. Therefore, exercises help mounding the tongue properly and clear the speech of the child.

## Conclusions

Ankyloglossia frequently leads to malocclusion, gingival recession, limited tongue mobility, difficulty speaking, and difficulties nursing or bottle-feeding infants. The gold standard for treating newborns with tongue-ties is a frenectomy, as it is a safe method. The surgical procedure is rapid and causes little pain as there aren't many blood vessels and nerve endings throughout the lingual frenulum. As a result of the quick advancements in laser technology and a better knowledge of how various laser systems interact with the human body, the use of lasers in dentistry has increased. Diode lasers are preferred by clinicians due to several benefits over using a scalpel blade. This helps the infant form healthy nursing habits from an early age. Patient's quality of life can be enhanced when they have appropriate lingual mobility. This is especially true if the issue is identified early on and can help avoid conditions that include crowding in dental arches, contractions in the palate, and breathing difficulties brought on by sleep.
